# Space-time clustering and associated risk factors of pulmonary tuberculosis in southwest China

**DOI:** 10.1186/s40249-018-0470-z

**Published:** 2018-08-17

**Authors:** Li Huang, Eniola Michael Abe, Xin-Xu Li, Robert Bergquist, Lin Xu, Jing-Bo Xue, Yao Ruan, Chun-Li Cao, Shi-Zhu Li

**Affiliations:** 1 Yunnan provincial Center for Disease Control and Prevention, Kunming, China; 2 National Institute of Parasitic Diseases, Chinese Center for Disease Control and Prevention, Ruijing Er road 207, Shanghai, 200025 China; 3 National Research Center for Tropical Disease, Shanghai, China; 4 Key Laboratory of Parasite and Vector Biology, Ministry of Health, Shanghai, China; 5 WHO Collaborating Center for Tropical Diseases, Shanghai, China; 6 Center for Drug Evaluation, China Food and Drug Administration, Beijing, China; 7 Ingerod, Brastad, Sweden

**Keywords:** Pulmonary tuberculosis, Spatio-temporal clustering, Regression analysis method, Risk factors, China

## Abstract

**Background:**

Pulmonary tuberculosis (PTB,both smear positive and smear negative) is an airborne infectious disease of major public health concern in China and other parts of the world where PTB endemicity is reported. This study aims at identifying PTB spatio-temporal clusters and associated risk factors in Zhaotong prefecture-level city, located in southwest China, where the PTB notification rate was higher than the average rate in the entire country.

**Methods:**

Space-time scan statistics were carried out using PTB registered data in the nationwide TB online registration system from 2011 to 2015, to identify spatial clusters. PTB patients diagnosed between October 2015 and February 2016 were selected and a structured questionnaire was administered to collect a set of variables that includes socio-economic status, behavioural characteristics, local environmental and biological characteristics. Based on the discovery of detailed town-level spatio-temporal PTB clusters, we divided selected subjects into two groups including the cases that resides within and outside identified clusters. Then, logistic regression analysis was applied comparing the results of variables between the two groups.

**Results:**

A total of 1508 subjects consented and participated in the survey. Clusters for PTB cases were identified in 38 towns distributed over south-western Zhaotong. Logistic regression analysis showed that history of chronic bronchitis (*OR* = 3.683, 95% *CI*: 2.180–6.223), living in an urban area (*OR* = 5.876, 95% *CI*: 2.381–14.502) and using coal as the main fuel (*OR* = 9.356, 95% *CI*: 5.620–15.576) were independently associated with clustering. While, not smoking (*OR* = 0.340, 95% *CI*: 0.137–0.843) is the protection factor of spatial clustering.

**Conclusions:**

We found PTB specially clustered in south-western Zhaotong. The strong associated factors influencing the PTB spatial cluster including: the history of chronic bronchitis, living in the urban area, smoking and the use of coal as the main fuel for cooking and heating. Therefore, efforts should be made to curtail these associated factors.

**Electronic supplementary material:**

The online version of this article (10.1186/s40249-018-0470-z) contains supplementary material, which is available to authorized users.

## Multilingual abstracts

Please see Additional file 1 for translations of the abstract into the five official working languages of the United Nations.

## Background

With an estimated 9.6 million infected people and 1.5 million deaths globally in 2014, tuberculosis (TB), caused by *Mycobacterium tuberculosis*, poses a serious challenge to global health [1]. Pulmonary tuberculosisis ranked second among the high-mortality infectious diseases and the World Health Organization (WHO) estimated 930 000, 1 million and 2.2 million new TB cases for China, Indonesia and India, respectively in 2015 [2]. Despite decreasing trend in incidence and mortality between 1990 and 2015, TB remains a serious threat to public health and social development in China [3, 4].

The burden of TB is particularly high across various regions of Yunnan, a mountainous and ethnically diverse province located in south-western China with poor economy. Pulmonary tuberculosis (PTB) ranks second among the notifiable infectious diseases prevalent in the area [5], and the Yunnan provincial surveillance system for TB reported recently that the overall incidence is 56 cases per 100 000 population [4]. Moreover, it was noted that TB incidence in some regions were higher than the average reported for the province. Especially the incidence in Zhaotong, a prefecture-level city, accounts for the highest number of PTB cases in Yunnan [6]. Therefore, we deemed it necessary to investigate the determinants that could potentially influence high PTB prevalence in this region.

The spatial distribution of TB has been widely investigated in China [7, 8] and several studies have shown that this infection has a heterogeneous distribution in space and time [9, 10]. However, information on the risk factors influencing TB aggregation is scarce [11–13]. PTB is known to be a chronic respiratory infectious disease transmitted from person to person, its distribution is associated with a large variety of factors in the human environment [14, 15]. Our previous study identified spatio-temporal clusters for the total PTB registration rate in Zhaotong [6], and this study aims to further this research by exploring the particular risk factors, so as to effectively provide both theoretical and technical support for TB prevention and control in China.

## Methods

### Study setting

The prefecture-level community of Zhaotong has a total population of 5.82 million including 0.54 million of ethnic minorities and covers an area of more than 23 000 km^2^ in north-east Yunnan Province. The terrain is low in the North and mountainous in the South. The lowest land is in Shuifu County (267 m above the mean sea level), while Qiaojia County represents the highest (4040 m). A total of 143 towns (132 rural towns and 11urban towns) in 11 counties were selected as the study sites.

### Recruitment of study subjects

Patients who had visited the Zhaotong Center for Disease Control (CDC) for PTB diagnosis between October 2015 and February 2016 were selected as the study subjects. PTB patients were consecutively included in the study according to the order of diagnosis.

The inclusion criteria were: (1) having active PTB (according to the new revision of the WS288–2008 [16] diagnostic manual); (2) willingness to participate in the study; and (3) having signed the informed consent form. Exclusion criteria included: (1) inability to cooperate with the investigators due to mental or physical disorders; and (2) refusal to sign the informed consent form for various reasons.

### Cluster investigation

Based on the number of PTB cases registered at the town level in Zhaotong, spatio-temporal analysis was used to identify and locate statistically significant aggregations of registered PTB from 2011 to 2015 in the nationwide TB online registration system. We applied SaTScan^TM^ software version 9.4.2 (https://www.satscan.org/) [17] as described in a previous study by our research group [6]. Furthermore, we divided PTB-diagnosed subjects registered between October 2015 and February 2016 into two groups: (1) cases living within towns identified as having spatial clusters; and (2) cases residing outside identified clusters (the control group). We linked/geo-referenced the location of PTB cases by their home addresses at town-level.

### Questionnaire approach

A structured questionnaire was developed and administered by the trained doctors from 11 county level CDCs engaging subjects in face to face interviews.

The questionnaire was divided into four sections including biological; behavioural; socio-economic and local environmental aspects, each section was further divided into sets of indicators as shown in Table 1 that includes full definitions of all variables investigated.

**Table 1:** Investigated potential indicators of PTB in Zhaotong

Biological aspects	Behavioural aspects	Socio-economic aspects	Local environment aspects
Age^a^	Alcohol intake^e^	Annual family income (yuan)^k^	Residence location^p^
Gender^b^	Chronic fatigue or overwork^f^	Below poverty level^c^	Family size^q^
BCG vaccination^c1^	Frequent Internet café visits^c^	CDC (km from residence)^l^	Number of persons per room^r^
BMI^d^	Marital status^g^	CDC (minutes from residence)^m^	Living space per person^s^
Hormone therapy^c^	Migrant work^c^	Educational level^n^	Windows per m^2^ of family space^t^
Cancer^c^	Smoking (active)^h^	Health insurance^c1^	Frequently keeping windows open ^c1^
Chronic bronchitis^c^	Smoking (passive)^i^	Occupation^o^	Coal used for cooking/heating^c^
Diabetes^c^	Spitting habit^c^		Electricity used for cooking/heating^c^
HIV/AIDS^c^			Firewood used for cooking/heating^c^
Kidney disease^c^			Natural/biogas used for cooking/heating^c^
Mental disease^c^			Other energy source for cooking/heating^c^
Parasitic disease^c^			PTB patient(s) living nearby^c^
Pneumoconiosis^c^			Contact with PTB patient(s)^c^
Other^c^			

*Abbreviations*: *AIDS* Acquired immune deficiency syndrome, *BMI* Body mass index, *CDC* Zhaotong Center for Disease Control, *HIV* Human immunodeficiency virus

Definitions: ^a^ <  15 = 1/15–29 = 2/30–44 = 3/45–59 = 4/≥60 = 5; ^b^male = 1/female = 0; ^c1^no = 1/yes = 0; ^c^yes = 1/no = 0,^d^ <  18.5 = 1/≥25 = 2/18.5–24.9 = 3; ^e^neve*r* = 1/in the past = 2/currently = 3; ^f^yes(light or heavy = 1/no = 0; ^g^unmarried = 1/married or remarried = 2/divorced or widowed = 3; ^h^never = 1/past smoker = 2/current smoker = 3; ^i^no exposure = 1/light exposure = 2/heavy exposure = 3; ^k^ <  2000 = 1/2000–5000 = 2/5001–10 000 = 3; ^l^ ≥ 10 001 = 4 <  15 = 1/15–30/31–60/≥61; ^m^ <  31 = 1/31–60 = 2/61–120 = 3/≥121; ^n^primary school or below = 1/middle or technical secondary school = 2/junior college and above = 3; ^o^farmer or migrant worker = 1/other = 2/unemployed = 3/student = 4; ^p^urban = 1/rural = 0; ^q^ <  3 = 1/3–4 = 2/5–6 = 3/> 7 = 4; ^r^ ≤ 1 = 1/1.6–2 = 2/> 2 = 3; ^s^ ≤ 10 m^2^ = 1/11–25 m^2^ = 2/26–50 m^2^ = 3/> 50 m^2^ = 4; ^t^low(< 0.05) = 1/medium(0.05–0.077) = 2/high(> 0.077) = 3

A total of 1508 PTB cases were investigated and all the questionnaires were completed as requested. All subjects signed the informed consent form and voluntarily participated in the survey.

### Data analysis

EpiData software, version 3.1 (http://www.epidata.dk/) was used to double-check the questionnaire data to avoid double entry. Data were analysed by SPSS version19.0 (https://www.ibm.com/products/spss-statistics) statistical software. The data were sorted out, then assigned binary variables (0 or 1) and transformed into multiple categorical variables by setting dummy variables. The characteristics of the biological, behavioural, socio-economic and environmental factors between PTB cases within and outside the spatial cluster identified were compared. Univariate logistic regression analysis was carried out and variables found to be significant were entered into a multivariate logistic regression model to analyse the association of factors of PTB spatial aggregation. The level indicating statistical significance for all analyses was set at 0.05.

## Results

### Reported PTB incidence in the study area

**Fig. 1:**
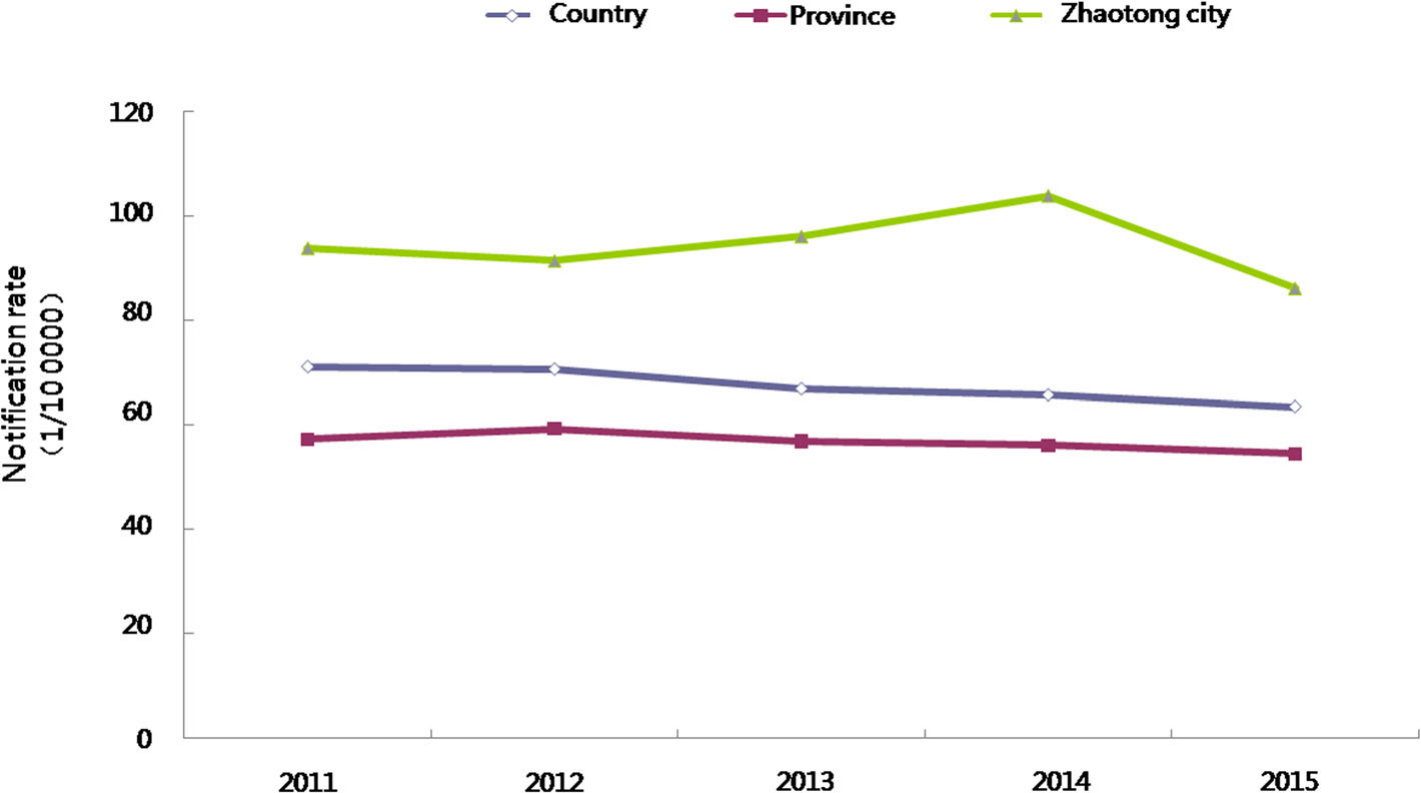
The temporal aspect: Notification rate of PTB in Zhaotong from 2011 to 2015

PTB notification rates per 100 000 population in Zhaotong were 93.8, 91.4, 96.0, 103.84 and 86.10, respectively between 2011 and 2015. As shown in Fig. 1, the average of these notification rates (90 per 100 000 population over the 5 years) studied exceeded the rate reported for the whole province and, indeed, the entire country.

### Spatio-temporal cluster analysis

Table 2 shows that there were five statistically significant clusters, each with a higher than expected numbers of cases, including one most likely cluster and four secondary ones. Altogether, they covered 38 towns (out of a total of 105) persisting for a long time (March 2013 to August 2015) with the most likely cluster involving 29 towns distributed over a large area of Zhenxiong County. The first secondary cluster involved Fenghuang Town covering three towns from April 2014 to June 2014; the second involved Dousha Town in Yanjin County covering three towns from February 2011 to July 2013; the third covered only one town, namely Cuihua Town and it persisted from March 2011 to October 2012, while the fourth involved Zhongming Town in Yiliang County covering two towns. The last secondary cluster emerged in December 2015 but lasted only for 1 month.

**Table 2:** Space-time PTB clusters in Zhaotong in the period 2011/1/1–2015/12/31

Cluster type	Time period	Centre and radius (km)	Areas covered (*n*)	Observed cases (*n*)	Expected cases (*n*)	RR^a^	*P*-value
Most likely cluster	2013/3/1–2015/8/31	Wufeng 45.51	29	5994	3418	2.01	< 0.001
Secondary cluster 1	2014/4/1–2014/6/30	Fenghuang 7.06	3	155	45	3.47	< 0.001
Secondary cluster 2	2011/2/1–2013/7/31	Dousha 13.76	3	362	214	1.70	< 0.001
Secondary cluster 3	2011/3/1–2012/10/31	Cuihua 0	1	119	58	2.06	< 0.001
Secondary cluster 4	2015/12/1–2015/12/31	Zhongming 9.04	2	23	6	3.91	0.030
Secondary cluster 5	2012/2/1–2012/5/31	Xiluodu 17.84	4	81	45	1.82	0.162
Secondary cluster 6	2012/1/1–2012/7/31	Baihetan 0	1	86	51	1.68	0.704

^a^Relative risk

### Characteristics of PTB cases within and outside the spatial clusters

Of the 1508 PTB cases investigated by questionnaire, 1038 were male (68.8%) and 470 female (31.2%). A significant proportion of the cases were found in the age groups 15–29 years old (> 30%) and 30–44 years old (around 25%). The proportion of farmers and migrant workers was 77.2%, students (8.4%), unemployed (2.9%), and others (5.9%). The treatment history showed that a great majority (1474) were receiving initial treatment (97.8%) with only 34 cases were under retreatment schedule (2.3%). More importantly, 813 cases (53.9%) of the 1508 subjects resided inside the spatial cluster, while the remaining 695 (46.1%) lived outside.

### Univariate regression analysis

#### PTB and biological factors

Location was used as the dependent variable with 14 indicators considered as independent variables.

There were no correlation between PTB and cancer and long-term treatment with hormones. However, as seen in Table 3, correlations with the other 12 variables were seen, in particular there were significant findings with regard to age group, BMI value, Bacillus Calmette-Guérin (BCG) vaccination, chronic bronchitis, co-infection with pneumoconiosis, as well as with a variety of other biological complications. Patients with chronic bronchitis, no BCG vaccination and those below 44 years of age had a strong association with PTB.

**Table 3:** Univariate analysis of PTB and biological factors

Variable	Group	Outside the cluster		Within the cluster		*χ* ^2^	*P-*value	*OR* (95% *CI*)
		No.	%	No.	%			
Age	< 15	9	1.3	31	3.8	12.431	0.000	4.054 (1.862–8.826)
	15–29	189	27.2	291	35.8	15.395	0.000	1.812 (1.347–2.439)
	30–44	164	23.6	212	26.1	7.031	0.008	1.521 (1.116–2.075)
	45–59	180	25.9	149	18.3	0.026	0.873	0.974 (0.708–1.340)
	≥ 60	153	22	130	16			1.000
Gender	Male	489	70.4	549	67.5	1.400	0.237	0.876 (0.704–1.091)
	Female	206	29.6	264	32.5			1.000
BCG vaccination	No	300	65.8	325	76.1	11.269	0.001	1.657 (1.234–2.225)
	Yes	156	34.2	102	23.9			1.000
BMI value	< 18.5	112	16.5	87	11	10.062	0.002	0.612 (0.452–0.829)
	≥ 25	54	7.9	50	6.3	2.359	0.125	0.730 (0.488–1.091)
	18.5–24.9	514	75.6	652	82.6			1.000
Chronic bronchitis	Yes	121	17.4	364	44.8	121.108	0.000	3.846 (3.025–4.888)
	No	574	82.6	449	55.2			1.000
Diabetes	Yes	19	2.7	14	1.7	1.761	0.184	0.623 (0.310–1.253)
	No	676	97.3	799	98.3			1.000
HIV/AIDS	Yes	3	0.4	1	0.1	1.186	0.276	0.284 (0.029–2.737)
	No	692	99.6	812	99.9			1.000
Kidney disease	Yes	1	0.1	4	0.5	1.214	0.271	3.431 (0.383–30.772)
	No	694	99.9	809	99.5			1.000
Mental disease	Yes	2	0.3	1	0.1	0.483	0.487	0.427 (0.039–4.716)
	No	693	99.7	812	99.9			1.000
Parasitic infection	Yes	0	0	2	0.3	0.000	0.999	0.000 (0.000–0.000)
	No	588	100	582	99.7			1.000
Pneumoconiosis	Yes	16	2.3	6	0.7	5.739	0.017	0.316 (0.123–0.811)
	No	679	97.7	807	99.3			1.000
Other bio-related complication(s)	Yes	125	18	69	8.5	28.942	0.000	0.423 (0.309–0.579)
	No	570	82	744	91.5			1.000

*HIV* Human immunodeficiency virus, *AIDS* Acquired immune deficiency syndrome

#### PTB and behavioural factors

Location was used as the dependent variable with eight indicators considered as independent variables.

Table 4 shows that there were significant differences in smoking, both active and passive, and history of alcohol intake. There was a particularly strong association between PTB and drinking alcohol as well as smoking, both by the patients and exposure to smoke in everyday life.

**Table 4:** Univariate analysis of PTB and behaviour characteristics

Variable	Group	Outside the cluster		Within the cluster		*χ* ^2^	*P-*value	*OR* (95% *CI*)
		No.	%	No.	%			
Alcohol intake	Never	532	76.5	651	80.1	2.853	0.091	0.697 (0.458–1.060)
	In the past	126	18.1	97	11.9	11.222	0.001	0.438 (0.270–0.710)
	Currently	37	5.3	65	8.0			1.000
Chronic fatigue (overwork)	Yes	537	77.3	653	80.3	2.100	0147	1.201 (0.937–1.538)
	No	158	22.7	160	19.7			1.000
Frequent visits to Internet cafés	Yes	68	9.8	77	9.5	0.042	0.837	0.965 (0.684–1.360)
	No	627	90.2	736	90.5			1.000
Marital status	Unmarried	134	19.3	193	23.7	0.001	0.974	0.990 (0.551–1.779)
	Married or remarried	539	77.6	588	72.3	1.031	0.310	0.750 (0.430–1.307)
	Divorced or widowed	22	3.2	32	3.9			1.000
Migrant labour history	Yes	218	31.4	269	33.1	0.507	0.476	1.082 (0.871–1.344)
	No	477	68.6	544	66.9			1.000
Smoking (active)	Never	384	55.3	501	61.6	0.003	0.953	0.991 (0.722–1.359)
	In the past	229	32.9	204	25.1	4.976	0.026	0.676 (0.480–0.954)
	Currently	82	11.8	108	13.3			1.000
Smoking (passive)	None	104	20.4	168	23.5	0.214	0.644	0.920 (0.647–1.309)
	Light	312	61.2	381	53.4	5.844	0.016	0.696 (0.518–0.934)
	Heavy	94	18.4	165	23.1			1.000
Spitting habit	Yes	224	32.2	285	35.1	1.337	0.248	1.135(0.916–1.407)
	No	471	67.8	528	64.9			1.000

#### PTB and socio-economic factors

Location was used as the dependent variable with seven indicators considered as independent variables.

Table 5 shows that there were significant PTB associations with family annual income, occupation and the time needed for the patients to reach CDC from their homes. Comparatively higher number of students with PTB were also found in the clustered areas.

**Table 5:** Univariate analysis of PTB and socio-economic factors

Variable	Group	Outside the cluster		Within the cluster		*χ* ^2^	*P-*value	*OR* (95% *CI*)
		No.	%	No.	%			
Annual family income (yuan)	< 2000	183	26.3	129	15.9	12.396	0.000	0.445 (0.283–0.698)
	2000–5000	295	42.4	422	51.9	0.232	0.630	0.902 (0.594–1.371)
	5001–10 000	176	25.3	197	24.2	2.398	0.122	0.706 (0.454–1.097)
	≥ 10 001	41	5.9	65	8			1.000
Below poverty level	Yes	131	19.6	147	20	0.038	0.845	1.027 (0.789–1.335)
	No	537	80.4	587	80			1.000
Educational level	Primary school and below	446	64.2	441	54.2	1.267	0.260	0.688 (0.359–1.320)
	Middle school	233	33.5	349	42.9	0.015	0.903	1.042 (0.539–2.015)
	Junior college and above	16	2.3	23	2.8			1.000
Occupation	Farmer or migrant worker	583	83.9	650	80	12.023	0.001	0.509 (0.347–0.746)
	Other work	41	5.9	54	6.6	3.335	0.068	0.601 (0.348–1.038)
	Unemployed	29	4.2	17	2.1	13.577	0.000	0.268 (0.133–0.540)
	Student	42	6	92	11.3			1.000
Medical insurance	No	9	1.3	7	0.9	0.663	0.415	0.662 (0.245–1.787)
	Yes	686	98.7	806	99.1			1.000
Distance from medical facility (km)	< 15	162	23.4	222	27.5	0.693	0.405	1.129 (0.849–1.502)
	15–30	150	21.7	161	20	0.646	0.421	0.884 (0.655–1.194)
	31–60	207	29.9	213	26.4	1.361	0.243	0.848 (0.642–1.119)
	≥ 61	173	25	210	26.1			1.000
Time to reach medical facility (min)	< 31	172	24.9	186	23	0.455	0.500	1.103 (0.829–1.469)
	31–60	148	21.4	156	19.3	0.228	0.633	1.076 (0.798–1.450)
	61–120	172	24.9	269	33.3	11.128	0.001	1.596 (1.213–2.100)
	≥ 121	200	28.9	196	24.3			1.000

#### PTB and environmental factors

Location was used as the dependent variable with seven indicators considered as independent variables.

As shown in Table 6, there were significant associations between PTB and place of residence, the type of fuel used for cooking and heating in the household and the amount of living space per person. Patients living in urban areas, using coal for household cooking and heating and a living space per person not more than 10 m^2^ had higher risk of PTB, while households using electricity for cooking and heating were associated with lower PTB risk.

**Table 6:** Univariate analysis of PTB and local environmental factors

Variable	Group	Outside the cluster		Within the cluster		*χ* ^2^	*P-*value	*OR* (95% *CI*)
		No.	%	No.	%			
Place of residence	Urban	46	6.6	92	11.3	9.727	0.002	1.800 (1.244–2.605)
	Rural	649	93.4	721	88.7			
Family size	< 3	72	10.4	60	7.4	2.431	0.119	0.676 (0.414–1.106)
	3–4	281	40.4	306	37.6	0.389	0.533	0.884 (0.600–1.303)
	5–6	286	41.2	378	46.5	0.128	0.721	1.073 (0.730–1.576)
	> 7	56	8.1	69	8.5			
Number of persons per room	≤ 1	238	34.4	255	32	0.907	0.341	0.886 (0.690–1.137)
	1.6–2	230	33.2	271	34	0.043	0.835	0.974 (0.759–1.250)
	> 2	224	32.4	271	34			
Living space per person (m^2^)	≤ 10	30	4.4	62	7.9	7.199	0.007	2.606 (1.294–5.246)
	11–25	371	54.6	459	58.5	2.387	0.122	1.560 (0.887–2.742)
	26–50	250	36.8	241	30.7	0.442	0.506	1.215 (0.684–2.160)
	> 50	29	4.3	23	2.9			
Number of windows per m^2^ of family space	Low	187	31.6	244	37.3	0.694	0.405	1.158 (0.820–1.637)
	Medium	318	53.7	313	47.8	0.649	0.420	0.874 (0.629–1.213)
	High	87	14.7	98	15			
Frequently keeping windows open	No	175	25.2	203	25	0.009	0.925	0.989 (0.783–1.249)
	Yes	520	74.8	610	75			
Coal used for cooking/heating	Yes	171	24.6	651	80.1	407.611	0.000	12.314 (9.650–15.713)
	No	524	75.4	162	19.9			
Electricity used for cooking/heating	Yes	394	56.7	125	15.4	254.639	0.000	0.139 (0.109–0.177)
	No	301	43.3	688	84.6			
Firewood used for cooking/heating	Yes	225	32.4	287	35.3	1.431	0.232	0.877 (0.708–1.087)
	No	470	67.6	526	64.7			
Natural- or biogas used for cooking/heating	Yes	14	2	56	6.9	17.808	0.000	3.598 (1.985–6.522)
	No	681	98	757	93.1			
Other energy source for cooking/heating	Yes	0	0	5	0.6	0.000	0.999	0.000 (0.000–0.000)
	No	695	100	808	99.4			
PTB patient(s) living nearby	Yes	74	17.6	85	19	0.266	0.606	1.095 (0.776–1.545)
	No	346	82.4	363	81			
Contact with PTB patient(s)	Yes	70	17.9	100	15.2	1.312	0.252	0.822 (0.588–1.149)
	No	320	82.1	556	84.8			

*PTB* Pulmonary tuberculosis

### Multivariate logistic regression analysis

Table 7 shows that a total of 17 independent variables were statistically significant in univariate logistic regression analyses, and were therefore subsequently entered into the multivariate logistic regression model. The outcome was that a long history of bronchitis (*OR* = 3.683, 95% *CI*: 2.180–6.223), living in an urban area (*OR* = 5.876, 95% *CI*: 2.38–14.502) and use of coal as the main fuel for domestic use (*OR* = 9.356, 95% *CI*: 5.620–15.576) were seen as independently associated with clustering. While a non-smoking habit (*OR* = 0.340, 95% *CI*: 0.137–0.843), using electricity as the main source for cooking and heating (*OR* = 0.209, 95% *CI*: 0.122–0.359) and a BMI below the norm (*OR* = 0.516, 95% *CI*: 0.275–0.968) were protective factors.

**Table 7:** Multivariate analysis of the PTB spatial cluster situation

Variable	*β*	*Sx*	*Waldx* ^*2*^	*P-*value	*aOR*	95% *CI*
Age			1.499	0.827		
< 15	0.791	0.797	0.987	0.320	2.207	0.463–10.513
15–29	0.289	0.351	0.678	0.410	1.335	0.671–2.654
30–44	0.311	0.342	0.825	0.364	1.365	0.698–2.669
45–59	0.134	0.341	0.155	0.694	1.144	0.586–2.232
≥ 60					1.000	
BMI value			5.236	0.073		
< 18.5	-0.663	0.322	4.246	0.039	0.516	0.275–0.968
≥ 25	-0.494	0.396	1.555	0.212	0.610	0.281–1.326
18.5–24.9					1.000	
BCG vaccination						
No	0.063	0.283	0.049	0.825	1.065	0.612–1.854
Yes					1.000	
Chronic bronchitis						
Yes	1.304	0.268	23.757	0.000	3.683	2.180–6.223
No					1.000	
Pneumoconiosis						
Yes	-0.441	1.174	0.141	0.707	0.644	0.065–6.422
No					1.000	
Other biology-related complication(s)						
Yes	-0.297	0.31	0.92	0.337	0.743	0.405–1.363
No					1.000	
Alcohol intake			1.767	0.413		
Never	0.002	0.558	0	0.997	1.002	0.335–2.993
In the past	-0.485	0.602	0.649	0.421	0.616	0.189–2.003
Currently					1.000	
Smoking (active)			5.798	0.055		
Never	-1.08	0.464	5.418	0.020	0.340	0.137–0.843
In the past	-1.059	0.477	4.932	0.026	0.347	0.136–0.883
Currently					1.000	
Smoking (passive)			0.85	0.654		
No exposure	-0.237	0.329	0.521	0.470	0.789	0.414–1.503
Light	-0.27	0.296	0.834	0.361	0.763	0.428–1.363
Heavy					1.000	
Annual family income (yuan)			3.263	0.353		
< 2000	-0.377	0.485	0.604	0.437	0.686	0.265–1.775
2000–5000	-0.244	0.421	0.335	0.563	0.784	0.344–1.788
5001–10000	-0.628	0.425	2.188	0.139	0.534	0.232–1.227
≥ 10001					1.000	
Occupation			2.382	0.497		
Farmer/migrant worker	-0.506	0.421	1.441	0.230	0.603	0.264–1.377
Other	-0.264	0.593	0.198	0.656	0.768	0.240–2.456
Unemployed	-0.864	0.621	1.934	0.164	0.421	0.125–1.424
Student					1.000	
Time to medical facility (min)			3.185	0.364		
< 31	0.55	0.346	2.53	0.112	1.733	0.880–3.412
31–60	0.072	0.317	0.051	0.821	1.074	0.577–1.999
61–120	0.302	0.278	1.179	0.277	1.352	0.784–2.330
≥ 121					1.000	
Place of residence						
Urban areas	1.771	0.461	14.758	0.000	5.876	2.381–14.502
Rural areas					1.000	
Coal used for cooking/heating						
Yes	2.236	0.26	73.923	0.000	9.356	5.620–15.576
No					1.000	
Electricity used for cooking/heating						
Yes	-1.564	0.275	32.247	0.000	0.209	0.122–0.359
No					1.000	
Natural- or biogas used for cooking/heating						
Yes	1.114	0.658	2.867	0.090	3.046	0.839–11.058
No					1.000	
Living space per person (m^2^)			1.643	0.650		
≤ 10	0.787	0.661	1.415	0.234	2.196	0.601–8.031
11–25	0.324	0.517	0.391	0.532	1.382	0.501–3.810
26–50	0.257	0.518	0.246	0.620	1.293	0.468–3.570
> 50					1.000	

*BMI* Body mass index, *BCG* Bacillus Calmette-Guérin

## Discussion

This study explore the factors influencing the spatial clustering of PTB previously found in this region [6]. We found that the distribution of PTB in Zhaotong is not random, but rather spatially clustered in south-western Zhaotong. To explore the factors influencing spatial clustering and thus be able to evaluate the risk for infection, PTB cases were investigated with regard to residence within and outside the spatial clusters. The finding that chronic bronchitis, living in an urban area, smoking and the use of coal as the main domestic fuel were strongly associated with PTB clustering, interventions should be targeted towards these factors.

Several studies have shown that smoking increases the risk of PTB infection [18, 19], Lin et al. [20] pointed out that smoking is an independent risk factor for PTB, which is not affected by alcoholism and other social factors. Tobacco smoke contains a variety of harmful substances, which can lead to the damage of respiratory epithelial cell cilia, which affects the body’s ability of remove inhaled *M. tuberculosis* by the macrophage phagocytosis [20]. Air contamination that arises from dust and chemical fumes produced by coal combustion impair the respiratory tract in a similar way. Chronic respiratory diseases is identified as an associated risk factor to PTB because it reduces the body immune function and breaks down the respiratory defence functions [21]. Indeed, the high incidence of chronic bronchitis in the region can be attributed to the use of coal for family cooking and heating. Findings from this study shows that the use of coal as the main source of fuel for cooking in the region has a unique influence on PTB spatial cluster in Zhaotong. The highest number of PTB cases here was registered during our study period, which coincides with a period of low temperatures. The dominant reliance on coal for cooking and heating coupled with other unhealthy indoor activities such as smoking expose the people to air pollution, which is exacerbated in the winter season due to poor ventilation. It has previously been shown that microscopic particulate matter (PM), a complex mixture that forms a critical portion of air pollution which has long been known to increase the risk of morbidity and mortality for many diseases, is strongly correlated with solid fuel usage for cooking and heating [22].

Supporting the reverse proof of air pollution with respect to PTB infection, our findings have shown that the use of electricity as the main household fuel for cooking and heating is not associated with the development of PTB. This buttress the fact that coal usage as fuel for cooking is unhealthy and increases the risk of PTB infection. Meanwhile, our findings does not indicate that the use of gas by households is a protective factor. This might be as a result of poor access to such means of cooking or heating, which makes it play an insignificant part in the analysis.

Living in urban areas was found to be an independent risk factor for PTB spatial cluster. Several studies from abroad reported similar findings. For example, TB clusters has been reported in surrounding shelters for homeless people in an urban centre at Texas, near a tuberculosis treatment centre in India, in urban and industrial areas of Japan and around major urban centres in Portugal [23]. Urbanization is closely associated with PTB aggregation. While urban areas tend to have many public large places, a large number of urban migrant population with PTB, may play crucial role in spreading the disease [24]. Li Tao et al. [25] reported that the PTB epidemic is mainly distributed in areas with high population density and frequent movement of people. Therefore, it is pertinent to continually strengthen PTB prevention and control programme activities, especially in the urban areas.

Several studies have reported that individuals with a low BMI have a higher risk of being infected with TB than those with normal BMI [26, 27]. This might be due to low cellular immunity, which has been shown to be associated with a low BMI [26].However, our findings do not agree with these reports, which might be as a result of the different classification standard for the BMI normal value. Meanwhile, we used the WHO standard for BMI classification, whose normal range is from 18.5 to 24.9. It might be due to a low proportion of patients with low BMI, such as students infected with TB and patients under 45 years old who were in the identified clusters compared with the group not in the clusters. Therefore, this subject needs to be explored in-depth before its application.

The relationship between socio-economic situation and TB has a long history. Previous studies confirmed that TB is closely attributed to poverty [18, 25]. Finding from our study showed that households’ annual income was only significant in univariate analysis and not in multivariate analysis after adjusting other factors. Firstly, the overall economic situation in Zhaotong is poor on average and the distribution of socio-economic situation in this region appears to be homogenous. Regardless they were within spatial clusters or outside spatial clusters, the PTB patients’ family annual income was not particularly high. Secondly, people in urban areas are richer than the rural areas, but have higher PTB rates, and urbanization turns out to be a confounding factor that weakens the relationship between poverty and PTB. In addition, the family annual income may be linked to other confounding factors, such as migrant population, physical exercise, mental stress, etc., thus not significant in the multivariate analysis.

Our study is based on a big sample size and high data quality, but the influencing factors discussed in this paper are not the only factors affecting PTB distribution. Factors that need further exploration are the nutritional status of patients and genetic factors. Firstly, the nutritional status of patients is not only associate with BMI values, but also the intake of various nutrients [28]. The deficiency of vitamin A has been suspected to contribute to PTB development [29, 30], while other studies stress the role vitamin D [31, 32]. Secondly, a number of genes and chromosomal regions that are associated with PTB susceptibility have been identified [27, 30], and they may play an important role in the susceptibility and degree of PTB severity [29, 33]. In addition, it is important to note that the number of cases in some of the risk factors group were too small to enable us establish the relationship between these factors and PTB aggregation. However, we plan to use larger sample sizes in our future studies as this would give us the opportunity to have increased number of cases in the risk groups and thus add statistical power to our calculations. Our data may have selection bias because the values of independent variables were PTB patients information and the social demographic variables in each location could be different from the values of PTB cases and general population. Despite these limitations, our study has provided useful information that will improve our understanding on the risk factors of PTB spatio-temporal clustering, not only in north-eastern Yunnan Province but also elsewhere.

## Conclusions

Spatial clustering analysis is an important tool that helps provide aetiological clues. We found PTB specially clustered in south-western Zhaotong. The strong associated factors influencing the PTB spatial cluster including: the history of chronic bronchitis, living in the urban area, smoking and the use of coal as the main fuel for cooking and heating. Therefore, interventions should be targeted towards these factors.

## Additional file

Additional file 1: Multilingual abstracts in the five official working languages of the United Nations. (PDF 240 kb)

## Abbreviations

AIDS: Acquired immune deficiency syndrome
a*OR*: Adjusted odds ratio
BCG: Bacillus Calmette-Guérin
BMI: Body mass index
CDC: Center of Disease Control and Prevention
*CI*: Confidence interval
HIV: Human immunodeficiency virus
*OR*: Odds ratio
PM: Particulate matter
PTB: Pulmonary tuberculosis
RR: Relative risk
TB: Tuberculosis
WHO: World Health Organization
