# Supplementary material for: Space-time clustering and associated risk factors of pulmonary tuberculosis in southwest China
Source: Infect Dis Poverty. 2018 Aug 17;7:91. doi: 10.1186/s40249-018-0470-z (PMC6097331; doi:10.1186/s40249-018-0470-z)
Supplement: Supplementary file 1 — Multilingual abstracts in the five official working languages of the United Nations. (PDF 240 kb) [file 40249_2018_470_MOESM1_ESM.pdf]

Translation of the abstract into the five official working languages of the United Nations

التكتل الزماني المكاني وعوامل المخاطرة المتعلقة بالسل الرئوي في جنوب غرب الصين

لي هوانغ، إنيولا مايكل إيب، شن-شو لي، روبرت بِركوِست ، لين شو، جين- بو شيا، ياو روان، تشوان-لي تشاو، شي-جو لي.

الملخص

الخلفية: السل الرئوي (PTB، بنوعيه إيجابي اللطخة وسلبي اللطخة) هو مرض معدي ينتقل جواً ويشكّل محل خوف كبير على الصحة العامة في الصين وأنحاء العالم الأخرى التي تم الإبلاغ عن توطّن السل الرئوي بها. تهدف هذه الدراسة إلى التعرّف على التكتلات الزمانية والمكانية لمرض السل الرئوي وعوامل الخطر المرتبطة به في تشاوتونج، وهي مدينة بمستوى مقاطعة تقع في جنوب غرب الصين كان معدل الإخطار بمرض السل الرئوي بها أعلى من المعدّل المتوسط في البلاد بِأسرها.

المنهجية: تم إجراء إحصائيات المسح الزمانية المكانية بالاستعانة ببيانات السل الرئوي المسجلة في نظام التسجيل الوطني الإلكتروني للسل من عام 2011 إلى 2015 لتحديد التجمعات المكانية، ووقع الاختيار على مرضى السل الرئوي الذين شُخصّت حالاتهم بين أكتوبر 2015 و فبراير 2016. بعد ذلك، أُجري لهم استبيان منتظم لجمع مجموعة من المتغيرات من بينها الحالة الاجتماعية والاقتصادية والخصائص السلوكية والخصائص البيولوجية والبيئية المحلية. وبناءً على تكتلات مرض السل الرئوي الزمانية والمكانية المفصّلة على مستوى المدن التي اكتشفناها، قسّمنا الأفراد المختارين إلى مجموعتين، بما فيهم الحالات التي تسكن داخل وخارج التكتلات المحدّدة. بعد ذلك، طبّقنا تحليل الانحدار اللوجستي، مع مقارنة نتائج المتغيرات بين كلتا المجموعتين.

النتائج: وافق على الاستبيان وشارك به ما بلغ مجموعه 1,580 فرداً. وتم التعرف على تكتلات لحالات السل الرئوي في 38 مدينة. أظهر تحليل الإنحدار اللوجستي أن تاريخ الإلتهاب الشعبي المزمن (نسبة الأرجحية = 3.683, 95% مجال الثقة: 2.10 -6.223) والحياة في المناطق الحضرية (نسبة الأرجحية= 5.876, 95% مجال الثقة: 2.381 -14.502) واستخدام الفحم كمصدر رئيسي للوقود (نسبة الأرجحية = 9.356, 95% مجال الثقة: 5.620 -15.576) كانوا مرتبطين بشكل مستقل بالتكتلات. في حين أن عدم التدخين (نسبة الأرجحية=0.340, 95% مجال الثقة: 0.137-0.843) هو عامل الوقاية في التكتل المكاني.

الاستنتاجات: بينت نتائج من هذه الدراسة وجود تكتلات مفصّلة على مستوى المدينة لمعدل تسجيل السل الرئوي، فضلاً عن أنها تشير إلى وجود ارتباط إيجابي بين تكتلات السل الرئوي المكانية التي تم تحديدها وكلّ من تاريخ الإلتهاب الشعبي المزمن، والحياة في المناطق الحضرية، والتدخين، واستخدام الفحم كمصدر رئيسي للوقود في الطهي والتدفئة. من ثم، لابد من بذل الجهود للحد من هذه العوامل المترابطة.

Translated from English version into Arabic by Susan Fegy and Heba Kandel, through

中国西南部地区肺结核空间聚集性影响因素研究

黄莉, Eniola Michael Abe, 李新旭, Robert Bergquist, 许琳, 薛静波, 阮瑶, 曹淳力, 李石柱

引言：肺结核（包括涂片阳性和阴性）是一种呼吸道传播疾病，作为重要的公共卫生问题受到中国和世界其它流行地区的关注。位于中国西南部的昭通市的肺结核报告发病率远远高于全国的平均水平，本研究旨在确定昭通市肺结核时空聚集区及其相关危险因素。
方法：利用全国结核病网络专报系统 2011～2015 年登记昭通市肺结核数据进行时空扫描统计，探测具有统计学意义的聚集乡镇。对昭通市 2015 年 10 月至 2016 年 2 月期间诊断的肺结核患者进行问卷调查，收集患者的社会经济地位、行为特征、环境和生物学特征变量信息。根据调查对象所处的乡镇，将其分为聚集区内和聚集区外两组，采用 Logistic 回归分析比较两组变量的差异，探讨肺结核聚集性分布的影响因素。
结果：共有 1508 名患者同意参与调查。肺结核在 38 个乡镇呈聚集性分布。Logistic 回归分析显示，慢性支气管炎病史（*OR*＝3.683，95% *CI*：2.180～6223），居住地为城区（*OR*＝5.876，95% *CI*：2.38～14502），以煤炭为主要燃料（*OR*＝9.356，95% *CI*：5.620—1557）与空间聚集区独立相关，而不吸烟（*OR*＝0.340，95% *CI*：0.137～0.843）是空间聚集区的保护因素。

结论：这项研究的结果显示了详细的乡镇级别肺结核聚集区，与空间聚集区独立相关的因素是患有慢性支气管炎、居住地在城区、吸烟、以煤炭为家庭做饭取暖主要燃料，因此，应努力减少这些相关因素。

Translated from English version into Chinese by Li Huang

Agrégats spatio-temporels et facteurs de risque associés de tuberculose pulmonaire dans lesud-ouest de la Chine

Li Huang, Eniola Michael Abe, Xin-Xu Li, Robert Bergquist,Lin Xu, Jing-Bo Xue, Yao Ruan, Chun-Li Cao et Shi-Zhu Li

Résumé

Contexte : La tuberculose pulmonaire (TP, à frottis positifs comme négatifs) est une maladie infectieuse aéroportée qui représente un problème majeur de santé publique en Chine et dans d'autres parties du monde où elle est endémique. La présente étude vise à identifier les agrégats spatio-temporels de TP à l'échelle de la préfecture de Zhaotong, dans le sud-ouest de la Chine, où le taux de déclaration de cas de TP est supérieur à la moyenne à l'échelle du pays.
Méthodes : Les statistiques d'analyse spatio-temporelles ont été collectées à partir des données enregistrées du réseau national d'enregistrement en ligne de la TP de 2011 à 2015, afin d'identifier les agrégats spatiaux. Des cas de tuberculose pulmonaire diagnostiqués entre octobre 2015 et février 2016 ont été sélectionnés et un questionnaire structuré a été administré aux patients afin de recueillir un ensemble de variables incluant le statut socio-économique, des caractéristiques de comportement, ainsi que des paramètres environnementaux et biologiques locaux. Après avoir découvert des agrégats spatio-temporels détaillés au niveau des agglomérations, nous avons séparé les sujets sélectionnés en deux groupes incluant les patients vivant à l'intérieur et à l'extérieur des agrégats identifiés. Nous avons ensuite procédé à une analyse de régression logistique pour comparer les résultats des variables entre les deux groupes.
Résultats : Au total, 1058 patients ont consenti à participer à l'enquête. Des agrégats de TP ont été identifiés dans 38 localités. L'analyse de régression logistique a montré que des antécédents de bronchite chronique (*OR* = 3,683, *IC* à 95 % de 2,180 à 6,223), le fait de résider en zone urbaine (*OR* = 5,876, *IC* à 95 % de 2,381 à 14,502) et l'utilisation de charbon comme principal combustible (*OR* = 9,356, *IC* à 95 % de 5,620 à 15,576) étaient associés indépendamment aux agrégats. En revanche, le fait de ne pas fumer (*OR* = 0,340, *IC* à 95 % de 0,137 à 0,843) était le principal facteur de protection contre ces risques agrégés.
Conclusions : Les résultats de cette étude ont révélé des agrégats détaillés de taux de signalement de la TP dans les localités et indiqué que les agrégats spatiaux de TP étaient associés à des antécédents de bronchite chronique, de vie en zone urbaine, de consommation de tabac et d'utilisation de charbon comme combustible principal pour la cuisine et le chauffage. Des mesures doivent donc être prises pour diminuer l'impact de ces facteurs de risque associés.

Translated from English version into French by Louis Gauvreau and Suzanne Assenat, through

TRANSLATORS
WITHOUT BORDERS

Пространственно-временная кластеризация и факторы риска, связанные с заболеваниями легочным туберкулезом, в юго-западных регионах Китая

Ли Хуан, Эниола Майкл Абэ, Синь-Сюй Ли, Роберт Бергквист, Линь Сюй, Цзин-Бо Сюэ, Яо Жуань, Чунь-Ли Цао и Ши-Чжу Ли

Аннотация

Обоснование: Легочная форма туберкулеза (ЛТБ, как с положительными, так и с отрицательными результатами микроскопии мазка мокроты) - это инфекционное заболевание с воздушно-капельным путем передачи, представляющее значительную проблему для здравоохранения в Китае и других частях мира, где отмечается эндемичность ЛТБ. Это исследование направлено на выявление пространственно-временных кластеров ЛТБ и связанных с ним факторов риска в городском округе Чжаотун, расположенном в юго-западной части Китая, где регистрируемая заболеваемость ЛТБ была выше, чем средний показатель по всей стране.
Методы: Для выявления кластеров был выполнен пространственно-временной анализ с использованием данных о заболеваемости ЛТБ, хранящихся в общенациональной электронной системе регистрации ТБ с 2011 по 2015 год. Были отобраны пациенты, у которых был установлен диагноз ЛТБ в период между октябрем 2015 г. и февралем 2016 г. С помощью структурированного опросника был получен набор переменных, который включал социально-экономический статус, поведенческие характеристики, а также характеристики местной окружающей среды и биологические характеристики. Отобранных субъектов разделили на две группы на основании выявления подробных пространственно-временных кластеров ЛТБ на уровне города. Эти группы включали случаи заболеваний у лиц, проживающих в границах определенных кластеров и за их пределами. Затем для сравнения результатов переменных между этими двумя группами применили логистический регрессионный анализ.
Результаты: В общей сложности 1508 субъектов дали согласие и приняли участие в опросе. Кластеры для случаев заболевания ЛТБ были определены в 38 городах. Логистический регрессионный анализ показал, что хронический бронхит в анамнезе (*ОШ [отношение шансов]*=3,683; 95%*ДИ [доверительный интервал]*: 2,180-6,223); проживание в городской зоне (*ОШ* = 5,876; 95%*ДИ*: 2,81-14,502) и использование угля в качестве основного топлива (*ОШ* = 9,356; 95%*ДИ*: 5,620-15,576) были независимо связаны с кластеризацией. При этом отсутствие курения (*ОШ* = 0,340; 95%*ДИ*: 0,137-0,843) является защитным фактором в пространственной кластеризации.
Выводы: Результаты этого исследования показали подробные кластеры регистрации случаев заболевания ЛТБ на уровне города, а также выявили, что установленные пространственные кластеры ЛТБ были положительно связаны с данными о хроническом бронхите в анамнезе, проживании в городских местностях, курением и использовании угля в качестве основного топлива для приготовления пищи и обогрева. Следовательно, необходимо прилагать усилия для уменьшения этих ассоциированных факторов.

Translated from English version into Russian by Ann Nosova and Natalia Potashnik, through

TRANSLATORS
WITHOUT BORDERS

Agrupación espacio-temporal y factores de riesgo asociados de tuberculosis pulmonar en el sudoeste de China

Li Huang, Eniola Michael Abe, Xin-Xu Li, Robert Bergquist,Lin Xu, Jing-Bo Xue, Yao Ruan, Chun-Li Cao and Shi-Zhu Li

Resumen
Antecedentes: La tuberculosis pulmonar (TP, tanto positiva como negativa) es una enfermedad infecciosa transmitida por el aire de gran preocupación para la salud pública en China y en otras partes del mundo donde la TP es endémica. El objetivo de este estudio es identificar las agrupaciones espacio-temporales de TP y los factores de riesgo asociados en la ciudad, a nivel de prefectura, de Zhaotong, en el suroeste de China, donde la tasa de notificación de TB fue superior a la tasa media de todo el país.
Métodos: Las estadísticas de escaneo espacio-temporal se llevaron a cabo utilizando datos de

pacientes aquejados de TBC registrados en el sistema nacional de registro en línea de la tuberculosis de 2011 a 2015, para identificar las agrupaciones espaciales. Se seleccionaron pacientes de TBC pulmonar diagnosticados entre octubre de 2015 y febrero de 2016 y se les entregó un cuestionario estructurado para recopilar un conjunto de variables que incluyen el nivel socioeconómico, las características de comportamiento y las características ambientales y biológicas locales. Basándonos en el descubrimiento de agrupaciones de TBC pulmonar, delimitadas a nivel ciudadano y espacio temporal, dividimos a los sujetos seleccionados en dos grupos, incluyendo los casos que residen dentro y fuera de los grupos identificados Después, se aplicó el análisis de regresión logística comparando los resultados de las variables entre los dos grupos.

Resultados: Un total de 1508 personas dieron su consentimiento y participaron en la encuesta. Se identificaron grupos de casos de TBC pulmonar en 38 ciudades. El análisis de regresión logística mostró que los antecedentes de bronquitis crónica(*OR*=3.683, 95％*CI*: 2.180-6.223), vivir en área urbana (*OR* = 5.876, 95％*CI*: 2.381-14.502) y usar carbón como combustible principal (*OR* = 9.356, 95％*CI*: 5.620-15.576) estaban asociados, de manera independiente, con la agrupación. Mientras que no fumar (*OR* = 0.340, 95％*CI*: 0.137-0.843) es el factor de protección de la agrupación espacial.

Conclusiones: Los resultados de este estudio han mostrado grupos detallados a nivel de ciudad de la tasa de registro de TBC pulmonar, e indican que los grupos espaciales de TBC pulmonar identificados estaban asociados positivamente con los antecedentes de bronquitis crónica, la vida en el área urbana, el tabaquismo y el uso del carbón como combustible principal para cocinar y calefacción. Por lo tanto, se deben hacer esfuerzos para reducir estos factores asociados.

Translated from English version into Spanish by Iria Carballo and Miguel Hernández, through

TRANSLATORS
WITHOUT BORDERS
